# Integration of GMR Sensors with Different Technologies

**DOI:** 10.3390/s16060939

**Published:** 2016-06-22

**Authors:** María-Dolores Cubells-Beltrán, Càndid Reig, Jordi Madrenas, Andrea De Marcellis, Joana Santos, Susana Cardoso, Paulo P. Freitas

**Affiliations:** 1 Department of Electronic Engineering, Universitat de València, Av. Universitat s/n, Burjassot 46100 , Spain; m.dolores.cubells@uv.es; 2 Department of Electronic Engineering, Universitat Politècnica de Catalunya, C. Jordi Girona, 1-3, Barcelona 08034, Spain; jordi.madrenas@upc.edu; 3 Department of Industrial and Information Engineering and Economics, University of L’Aquila, L’Aquila 67100, Italy; andrea.demarcellis@univaq.it; 4 INESC Microsistemas e Nanotecnologias, Rua Alves Redol 9, Lisbon 1000-029, Portugal; joana.dias.santos@ist.utl.pt (J.S.); scardoso@inesc-mn.pt (S.C.); pfreitas@inesc-mn.pt (P.P.F.)

**Keywords:** GMR, integration, technology

## Abstract

Less than thirty years after the giant magnetoresistance (GMR) effect was described, GMR sensors are the preferred choice in many applications demanding the measurement of low magnetic fields in small volumes. This rapid deployment from theoretical basis to market and state-of-the-art applications can be explained by the combination of excellent inherent properties with the feasibility of fabrication, allowing the real integration with many other standard technologies. In this paper, we present a review focusing on how this capability of integration has allowed the improvement of the inherent capabilities and, therefore, the range of application of GMR sensors. After briefly describing the phenomenological basis, we deal on the benefits of low temperature deposition techniques regarding the integration of GMR sensors with flexible (plastic) substrates and pre-processed CMOS chips. In this way, the limit of detection can be improved by means of bettering the sensitivity or reducing the noise. We also report on novel fields of application of GMR sensors by the recapitulation of a number of cases of success of their integration with different heterogeneous complementary elements. We finally describe three fully functional systems, two of them in the bio-technology world, as the proof of how the integrability has been instrumental in the meteoric development of GMR sensors and their applications.

## 1. Introduction

The giant magnetoresistance (GMR) effect was fully described in 1988 by A. Fert [1] and 1989 by P. Grunberg [2]. Both were granted with the Nobel Prize of Physics for this contribution in 2007. Basically, a GMR structure displays a change in its resistance as a function of the external magnetic field. Multilayered structures including ferromagnetic layers and nonmagnetic spacers can be engineered with optimized GMR response. In this way, GMR based devices were initially used in the read heads of magnetic massive data storage systems (hard disks drives) taking advantage of the binary high/low resistance states. Nowadays, GMR based sensors are well established, initially emerging by exploiting the linear range between both limit states. They have been successfully used in a huge range of scenarios demanding small devices with good figures of merit.

GMR structures display sensitivities that exceed those of their counterpart anisotropic magnetoresistance (AMR) or Hall sensors. GMR structures can be deposited and patterned into resistive strips with nominal resistance parameters that can be tuned in a huge range of values, which can be matched to different requirements. This flexibility allows the design of sensors with different arrangements: single elements, bridges, arrays ... Then, the generated electrical signal can be acquired by making use of well known electronic front-ends. The fabrication of GMR devices can be carried out by means of traditional deposition (e.g., sputtering) and UV lithography systems, which are available in small laboratories as well as in big companies. In this way, GMR sensors are, nowadays, the preferred option when the measurement of low magnetic fields in small spaces is required.

In this sense, GMR sensors have been successfully applied in many different environments, including: compass, automotive applications, angle measurement, encoding, detection of metallic bodies (also weapons), electrical current measurement, non destructive testing by means of eddy currents measurement, bioanalytes detection (molecules, viruses, cells ... by means of micro-labelling with magnetic beads), fluid control ... A good review is given in [3].

This rapid deployment from theoretical basis to market and state-of-the-art applications can not be explained solely by taking into account the figures of merit of GMR structures and related devices. In fact, in the last years, the GMR technology has been continuously demonstrating its capability of integration, also monolithically, with a wide range of complementary technologies, which has allowed the enhancement of its intrinsic properties as well as the spreading of its range of application. Among these accompanying technologies, we should highlight standard complementary metal-oxide-semiconductor (CMOS) technologies and microfluidics as the more relevant. In the former, GMR sensors have been successfully integrated within the chips including the microelectronics circuits (biasing, amplification, addressing ...), so providing compact sensing probes with better performance. In the latter, mainly in bio-technology applications, the bio-analytes have been driven closer to the sensing area, so enlarging the sensitivity and lowering the required amount of the samples.

Then, current challenges related with GMR technology focus on the definition of novel processes and their improvement for integrating GMR structures, with both general goals: to improve their intrinsically good properties or to spread their range of usage towards novel applications.

In this paper, we will collect the state-of-the-art advances of the integration of GMR sensors with different heterogenic technologies.

## 2. GMR Principles

### 2.1. GMR Fundamentals

**Figure 1:**
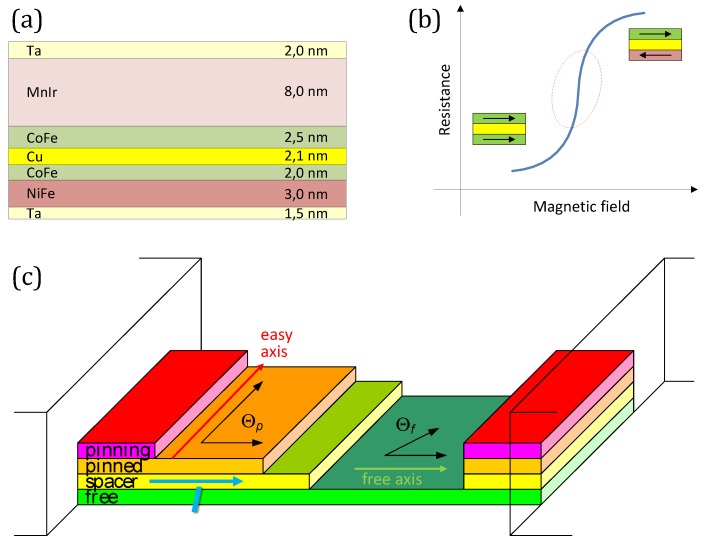
Description of the function mechanism in spin valves.

Functional GMR structures are usually composed by multi-layered engineered structures based on nanometric to sub-nanometric thick magnetic layers separated by a non-magnetic spacer. A typical structure, is detailed in Figure 1a. The relative orientation of the magnetization moments of the magnetic layers surrounding a thin non-magnetic layer strongly affects the electric current flowing in such multilayers. In fact, the resistance of the magnetic multilayer is low when the magnetizations of the magnetic layers are parallel but higher when the magnetizations are antiparallel, as depicted in Figure 1b. This is due to the spin-dependent scattering. The magnetoresistance (MR) ratio is usually defined as:

(1) $$\frac{\Delta R}{R}=\frac{R^{↑↓}-R^{↑↑}}{R^{↑↑}}$$

There are several kind of structures that can display GMR effect but, for engineered applications, multilayer structures are preferred due to their integration feasibility. Typical multilayered structures consist of two or more magnetic layers of a Fe-Co-Ni alloy, as can be permalloy, separated by a very thin non magnetic conductive layer, as can be Cu. By considering nanometric layers thickness, magnetic coupling between layers is slightly small. With this configurations, MR levels of about 4%–9% are achieved, with linear ranges of about 5 mT [3], which are good for sensing applications. The figures of merit of these devices can be improved by continuously repeating the basic structure in a sandwich fashion.

Spin valves are a particular configuration of a sandwich structure. In spin valves, an additional antiferromagnetic (*pinning*) layer is added to the *top* or *bottom* part of the structure, as shown in Figure 1c. In this sort of structures, there is no need of an external magnetic excitation to get the antiparallel alignment. In spite of this, the pinned direction (*easy axis*) is usually fixed by raising the temperature above the knee temperature (at which the antiferromagnetic coupling disappears) and then cooling it within a fixing magnetic field. Obviously, so obtained devices have a temperature limitation below the knee temperature. Typical values displayed by spin valves are a MR of 4%–20% with saturation fields of 1–8 mT [3].

For linear applications, and without excitation, pinned (easy axis) and free layers are preferably arranged in a crossed axis configuration (at $90^{\circ }$), as depicted in Figure 1c. In this way, the linear range, highlighted in Figure 1b, is improved and the sign of the external field is detected without the need of an additional magnetic biasing. The response this structure is given by [4]:

(2) $$\Delta R=\frac{1}{2}\left(\frac{\Delta R}{R}\right)R_{□}\frac{iW}{h}\cos\left(\Theta_{p}-\Theta_{f}\right)$$

where (ΔR/R) is the maximum MR level, $R_{□}$ is the sensor sheet resistance (15–20 Ω/□), *L* is the length of the element, *W* is its width, *h* is the thickness, *i* is the sensor current, and $\Theta_{p}$ and $\Theta_{f}$ are the angle of the magnetization angle of pinned and free layers, respectively. Assuming uniform magnetization for the free and pinned layers, for a linearized output, $\Theta_{p}=\pi /2$ and $\Theta_{f}=0$.

Giant magnetoresistance can also find in other structures. We collect three illustrative examples. Arana *et al.* [5] present a study on a contact-less position sensor based on a magnetoresistive thin film of Ag-Co alloy. Pena *et al.* [6] report on giant magnetoresistance in ferromagnet/superconductor superlattices. On the other hand, Pullini *et al.* [7] describe GMR in multilayered nanowires. In any case, a magnetic/non-magnetic interface is required in order to allow the spin-electron scattering producing the effect.

For real applications, the performance of GMR based devices can only be estimated when compared with their intrinsic noise sources. The noise power spectrum density (PSD) is commonly given in $V^{2}/\mathrm{Hz}$. Often, is much more convenient to use the amplitude spectrum density (ASD), expressed in $V/\sqrt{\mathrm{Hz}}$ for comparison with voltage signals. The sensitivity for a magnetoresistance signal, $S_{V}$ is usually given in V/V/T (V/V/A in current sensing applications). Typical values for GMR sensors are 20–40 V/V/T, e.g., 20–40 nV/nT when they are biased with 1 V. For comparing different sensors, it is recommendable to use the field equivalent noise power spectra density, sometimes called *detectivity*. It corresponds to the PSD divided by the sensitivity. For example, if a sensor displays a noise of 10 nV/$\sqrt{\mathrm{Hz}}$ at a given frequency and a sensitivity of 25 V/V/T, its detectivity will be 400 pT for 1 V bias.

The most relevant electronic noise mechanism in GMR based devices is the thermal noise (also called Johnson-Nyquist noise or white noise), which is directly related to the resistance of the sensor. It is a *white* noise, so it is independent of the frequency. It was first observed by Johnson [8] and interpreted by Nyquist [9]. It is expressed as:

(3) $$S_{V}\left(\omega \right)=\sqrt{4Rk_{B}T}$$

where *R* is the sensor resistance, $k_{B}$ is the Boltzmann constant and *T* is the temperature. For example, a 1 kΩ resistor at room temperature has 4 nV/$\sqrt{\mathrm{Hz}}$.

GMR devices also display 1/f noise (also called “pink” noise or Flicker noise). Its origin is on resistance fluctuations. It can only revealed by applying a current into the sensor. Its dependence with the frequency is described by the following phenomenological formula:

(4) $$S_{V}\left(\omega \right)=\frac{\gamma_{H}R^{2}I^{2}}{N_{C}f^{\beta }}$$

where $\gamma_{H}$ is a dimensionless constant proposed by Hooge [10], *R* is the sensor resistance, *I* is the bias current, $N_{C}$ is the number of current carriers, *f* is the frequency and *β* is an exponent typically in the order of 1.1/*f* noise can exhibit a non magnetic and a magnetic component with possible different slopes. The size and the shape of the sensors have a strong effect on the 1/*f* noise. Due to its average nature, and as followed by Equation (4), small GMR sensors display more 1/*f* noise than bigger ones. By considering equally thin sensors, the 1/*f* noise is roughly inversely proportional to their area [3].

**Figure 2:**
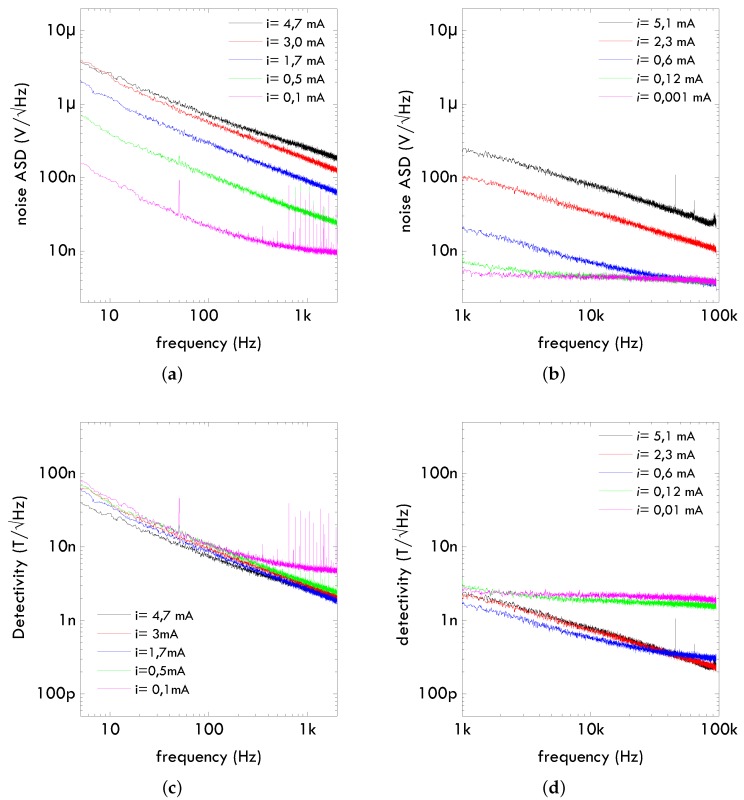
Noise measurement data on 3 × 200 μm${}^{2}$ spin valves as described in [11]: (**a**) low frequency noise; (**b**) high frequency noise; (**c**) low frequency detectivity; (**d**) high frequency detectivity.

As a representative example, we will give noise data on spin valves based on the multilayered structure depicted in Figure 1a. Measured sensitivity was 20 mV/mT (1 mA bias). Measured bandwidth was above 1 MHz [11]. The measured noise is shown in Figure 2a,b. The 1/f behaviour is clearly observed and the thermal noise limit well defined. If we take into account the measured sensitivity, we can draw the detectivity understood as the field equivalent noise, that is drawn in Figure 2c,d. The benefits of the frequency is clearly stated. The increase of the bias current has an impact on the field detectivity at higher frequencies, but there is no effect in the 1/f regime [3].

### 2.2. Deposition Issues

From structures like those previously described, GMR sensors can be fabricated by following a sort of techniques in a similar manner to those related to standard CMOS processes. For the deposition of functional structures, both cathodic sputtering and ion beam deposition methods are commonly used [3], with comparable results. It should be mentioned that a magnetic field must be applied during the deposition in order to give them the proper sensing capability. In addition, some studies have also been reported on the electrodeposition of GMR multilayers [12]. The patterning of the structures is usually carried out by UV lithography (with possibility of direct-writing and lift-off approaches), but electron beam lithography can also be used for obtaining sub-micron structures (2000 × 100 nm${\;}^{2}$, as reported in [13]). Because ionic doping (diffusion and implantation) is not required, GMR fabrication only involves low temperature processes. In fact, the higher temperature achieved during the fabrication of GMR based devices is during the post-annealing process that sometimes is required to set the interfacial exchange coupling between the antiferromagnetic and the reference layers (MnIr/CoFe) (usually below 300${\;}^{\circ }$). In this way, GMR sensors can be theoretically deposited onto low melting point substrates, as well as onto substrates including other kind of preprocessed structures. Such capability was demonstrated at the beginning, with a comparative study of GMR structures performance onto different substrates including glass, silicon, SiO${\;}_{2}$-Si substrates and polyimide or other flexible structures [14].

**Figure 3:**
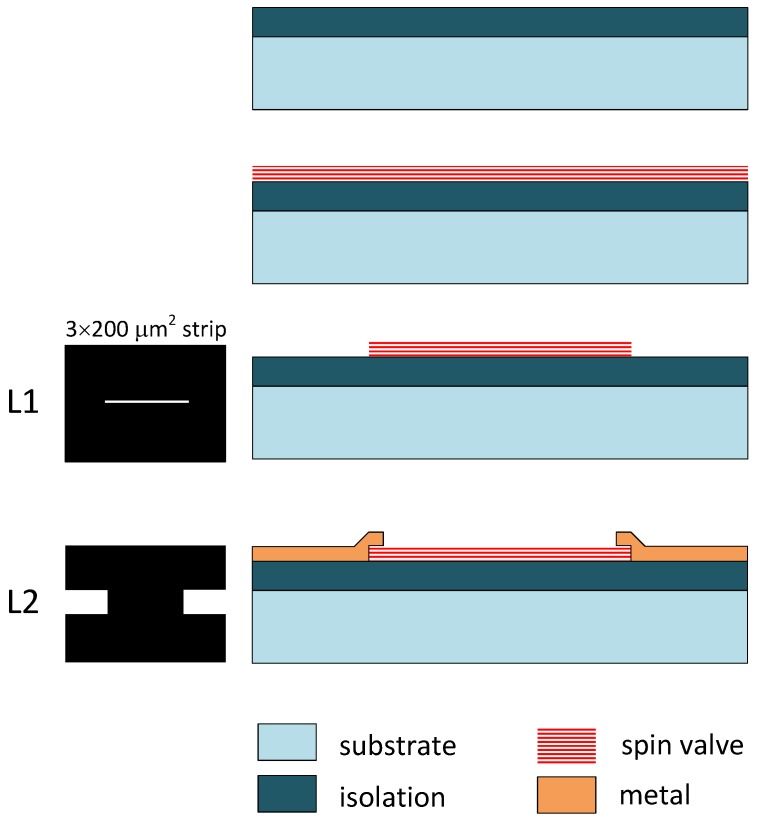
Basic fabrication process of a GMR elemental sensor.

In order to have functional devices, GMR multilayered structures have to be patterned into elements with proper resistance values for being used as sensors (usually L×W strips/rectangles). Then, these elements need to be contacted. Because of their particular interest we will come into detail on the typical fabrication process of spin-valve (SV) structures. For implementing a SV based device, only one lithographic step is required for patterning the structures (L1), and then another to design the contacts (L2), at the ends of the SV strip, as shown in Figure 3. The sheet resistance is inherent to the specific SV structure, but the final resistance value can be tuned by properly setting *L* and *W* of the strip. Usually, the minimum *W* value is constrained by the lithography resolution, and then the *L* value is obtained. For spin valve structures such as those described in the previous paragraph, devices of 200 μm × 3 μm give nominal resistances in the order of 1 kΩ.

#### 2.2.1. GMR Structures onto Flexible Substrates

With the requirements of actual applications, recent research efforts have focused on the deployment of GMR sensors onto flexible substrates, mainly organic polymers.

**Figure 4:**
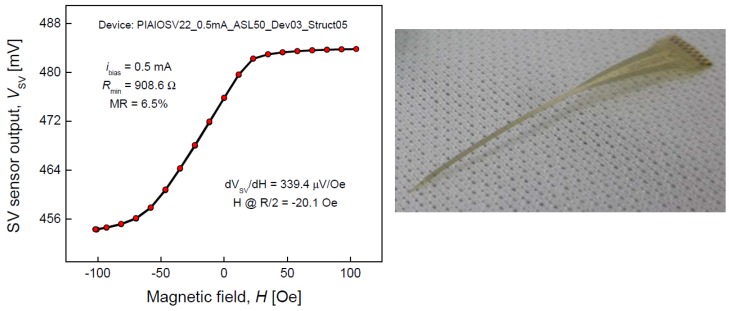
Spin valve devices deposited on polyimide as described in [17].

The more straightforward method for obtaining flexible GMR structures is by direct deposition onto flexible substrates. This was early demonstrated by Parkin *et al.* by depositing Co/Cu multilayers by DC sputtering onto 25 μm thick and 25 mm diameter kapton disks, with obtained MR levels exceeding 30% at room temperature [15]. Such studies were then spread to exchange biased sandwich (EBS) structures onto other substrates (mylar, transparency, kapton and ultem) as well as onto silicon substrates buffered with spin coated layers (polystyrene, polypropylene and 2-vinyl pyridine) [16]. So obtained structures displayed similar performances to those deposited onto rigid substrates. Also the INESC-MN laboratory has reported on successful spin valve sensors deposited onto polyimide substrates, with the characteristics detailed in Figure 4 [17].

**Figure 5:**
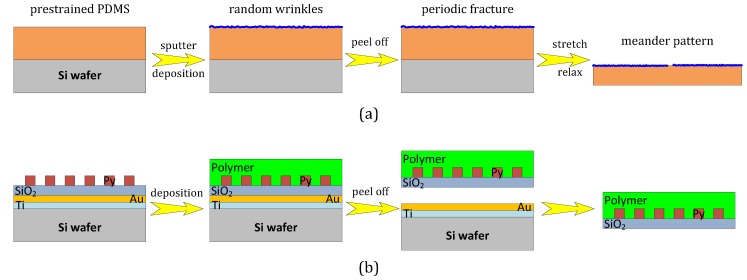
Different approaches for obtaining GMR structures onto flexible substrates: (**a**) as described in [19]; (**b**) as described in [20].

Buffer layers can be used for reducing the roughness of the plastic substrates, so improving the response of the deposited structures. A Ta layer (such as that displayed in Figure 3) can be used for this purpose. Photoresist layers, which are able to confer stretchability to the structure, can also being considered [18]. In this case, the photoresist layer can be then peeled off (with the deposited GMR structures) so obtaining a flexible and stretchable structure (see Figure 5a, [19]). In this work, a thick layer of poly(dimethylsiloxane) (PDMS), with a surface roughness <0.5 nm was spin coated onto a Si substrate (with a thin intermediate photoresist layer, for assisting the peeling). After patterning, a spin valve structure (Ta(2 nm)/IrMn(5 nm)/[Py(4 nm)/CoFe(1 nm)]/Cu(1.8 nm)/[CoFe(1 nm)/Py(4 nm)], Py standing for the Ni${\;}_{81}$Fe${\;}_{19}$ permalloy) was deposited by magnetron sputtering. After the lithographic lift-off process, the PDMS film was peeled from the rigid silicon wafer, by means of the antistick layer, leading to a free-standing elastic membrane covered with the lithographically structured magnetic film.

A similar pathway is described in [20] (see Figure 5b). In this case, permalloy nanostructures were patterned by electron beam lithography onto a “donor” Si substrate, with a previously sputtered tri-layer (Ti(3 nm)/Au(20 nm)/SiO${\;}_{2}$(t)). Then, a thin layer of the desired polymer was spin coated over the structures and cured at room temperature. Finally, a simple immersion of the chip in water was sufficient for allowing the mechanical lifting of the polymer layer incorporating the magnetic nanostrucutes.

Printing magnetic sensors is a promising and challenging way for obtaining low-cost devices onto different substrates (paper, polymer or ceramic based materials). First attempts are reported in [21]. In this work, a magnetic sensitive ink is prepared from previously deposited Co/Cu structures. It is successfully printed onto paper and Si/SiO${\;}_{2}$ substrates, giving magnetoresistance levels higher than 4% (measured upon high field saturation, at 2T). The demonstration of real functional devices is reported in [22].

A recent review of the integration of magnetoresistive devices on flexible/bendable media can be found in [23].

#### 2.2.2. GMR Structures onto Pre-Processed CMOS

Monolithic integration of GMR sensors with integrated circuits opens a wide range of cutting edge applications including, for example, bioengineering or spin microelectronics, which usually require a large number of connections and sensors. Fabrication techniques associated to GMR sensors are compatible with standard CMOS technologies, as initially demonstrated by NonVolatile Electronics (NVE) by using a dedicated 1.5 μm BiCMOS technology [24]. Later, Han *et al.* used chips made by 0.25 μm National Semiconductor Corporation (NSC) BiCMOS technology [25]. The compatibility with other semi-custom CMOS technologies such as the CN25 from the National Centre of Microelectronics in Spain has also been reported [26].

**Figure 6:**
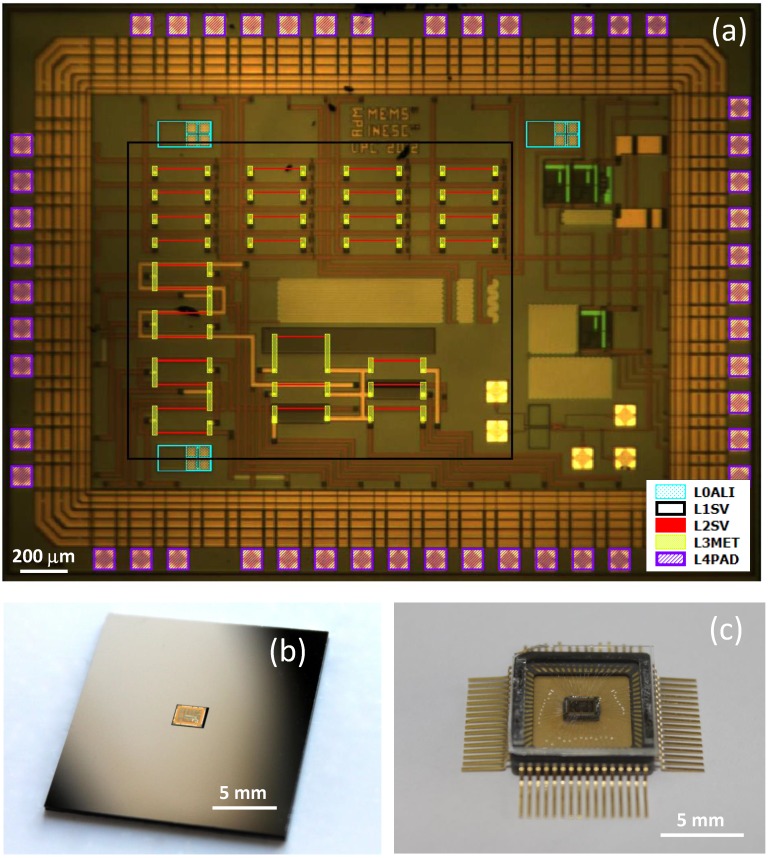
GMR based current sensors integrated with standard 0.35 μm–3.3V–AMS (2.5 × 1.5 mm${}^{2}$) chips. (**a**) Lithography masks for monolithic deposition of the spin-valve sensors; (**b**) Engineered sample holder substrate with the specifically machined hole; (**c**) Final encapsulated chip, after release from the holder.

For state-of-the-art applications, or pre-industrial demonstrators, which require low number of chips, it is advantageous to make use of standard non-dedicated general purpose CMOS technologies. In this respect, the INESC developed a method for depositing GMR structures onto dies from standard CMOS foundries [27]. Chips with dimensions 2.22 × 1.9 × 0.5 mm${\;}^{3}$ from 0.35 μm–3.3 V–AustriaMicroSystems (AMS) technology were used. Previously to the microfabrication process of the sensors, 1 × 1 in${\;}^{2}$ chip holders were fabricated onto a silicon substrate where a cavity with the chip dimensions and thickness was etched by deep reactive ion etching (DRIE). Then, the chip was glued on the cavity, using ultra-low viscosity glue. In this way, an easy-to-handle chip was engineered, enabling the processing of the GMR structures by using the standard microfabrication techniques without debonding. In this way, a 16 elements matrix including the biasing and the readout blocks was implemented for bio-applications. The magnetoresistance level of so deposited structures was similar to those deposited onto standard substrates. A 256 pixel magnetoresistive biosensor microarray in a 0.18 μm technology is reported in [28]. The same approach was used in [29] for integrating full Wheatstone bridges into 0.35 μm–3.3 V–AMS (2.5 × 1.5 mm${\;}^{2}$) chips including *R*-to-time converters [30] for integrated current monitoring. For such a post-process, five lithographic steps were carried out as depicted in Figure 6 and detailed in [29].

Finally, the flip-chip-based integration of GMR spin torque oscillators (STO) onto RF CMOS integrated circuits has been reported in [31], by following a chip-on-board (CoB) approach, resulting in a GMR STO + CMOS IC pair working in the 8–16 GHz rang with 1.5 nV/$\sqrt{\mathrm{Hz}}$.

## 3. Spreading the Performance

Being the favourite choice for many applications requiring the measurement of very low magnetic fields at the micron scale, GMR sensors still display some well known limitations such as thermal drifts, frequency bandwidth, hysteresis or limit of detectivity [32]. The most of them are intrinsic to the magnetoresistance mechanism, but they can be, in some cases, reduced by taking advantage of complementary technologies.

### 3.1. Thermal Drifts

GMR sensors are resistive elements, so they are affected by thermal variations, quantified by the corresponding temperature coefficient (TCR) [33]. It has been demonstrated that these effects can be reduced by biasing the sensors with current [3], by arranging the sensors in a bridge configuration [34] or by designing external compensation circuitry [35].

In [35], a ruthenium (Ru) meandered thermistor was deposited together with a engineered spin-valve based full bridge sensor. It was measured a TCR of 0.11%/${\;}^{\circ }$C for the spin-valve resistors and 0.16%/${\;}^{\circ }$C for the Ru thermistor. This element was used as a reference in the feedback loop current-source-based compensation circuit, so reducing in a factor 20 the thermal drift of the sensitivity in a 70 ${\;}^{\circ }$C range.

### 3.2. Limit of Detection

The sensitivity of a GMR sensor is intrinsically constrained to the magnetoresistance (MR) level of its multilayered structure and the linear range of operation, which is controlled by the anisotropy and demagnetizing fields at the free layer [4]. On the other hand, GMR sensors are typically affected by 1/*f* noise, which is a real physical handicap. The use of complementary technologies can overcome these limitations.

#### 3.2.1. Improving the Sensitivity

**Figure 7:**
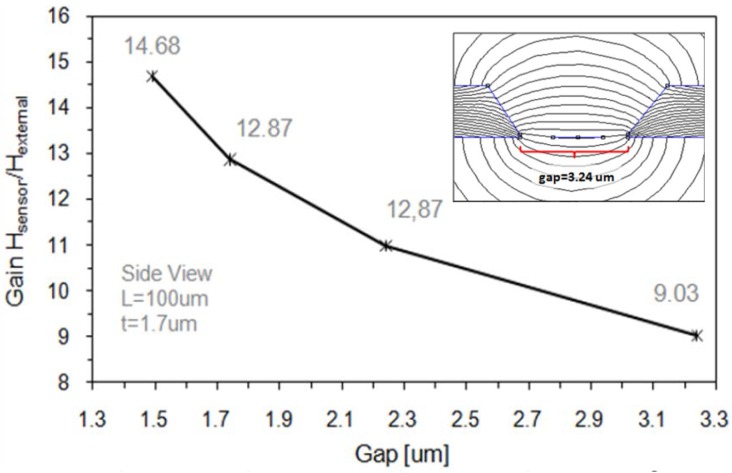
Simulated gain as a function of the gap between MFCs.

By decreasing the linear range of a GMR sensor its sensitivity is improved (the slope of the linear range of the response is increased, as illustrated in Figure 1b). When GMR multilayers are optimized, we can still use magnetic flux concentrators (MFC). In this sense, high permeability materials ($\mu_{r}\gg 1$) of 100 to 500 nm thickness can be used as MFC. MCF are capable of increasing the magnetic flux density near the sensing elements in a factor up to 100. This gain is determined by the overall geometry, the separation between poles and the corresponding $\mu_{r}$ [36]. For a proper optimized design, numerical simulations are often used [37]. As an example, in Figure 7 we show the simulated gain as a function of the gap between MFC.

Several materials have been successfully incorporated into the fabrication process of MFCs with GMR sensors. They have to be carefully chosen in order to avoid the introduction of undesirable effects such as hysteresis or additional noise. In this sense, with the use of optimized CoZrNb (350 nm thick) MFC, the improvement of the field detectivity from 1.3 nT$/\sqrt{\mathrm{Hz}}$ (no MFC) to 64 pT$/\sqrt{\mathrm{Hz}}$ at 100 kHz (gain factor of 20) has been demonstrated, with no added excess voltage noise [36].

It must be mentioned that the deposition of magnetic materials can be also used for implementing fixed biasing fields, which reduce the linear range, so enhancing the sensitivity in a factor up to 100 as demonstrated in [36]. The achieved high frequency detectivity was 2 pT$/\sqrt{\mathrm{Hz}}$. Moreover, in [38] a MFC is used for implementing a three axis vector magnetometer based on two sets of dual-bridge GMR sensors, with field noise espectral densities at 1 Hz between 3 and 9 nT$/\sqrt{\mathrm{Hz}}$ for all three sensing axes.

#### 3.2.2. Reducing the Noise

**Figure 8:**
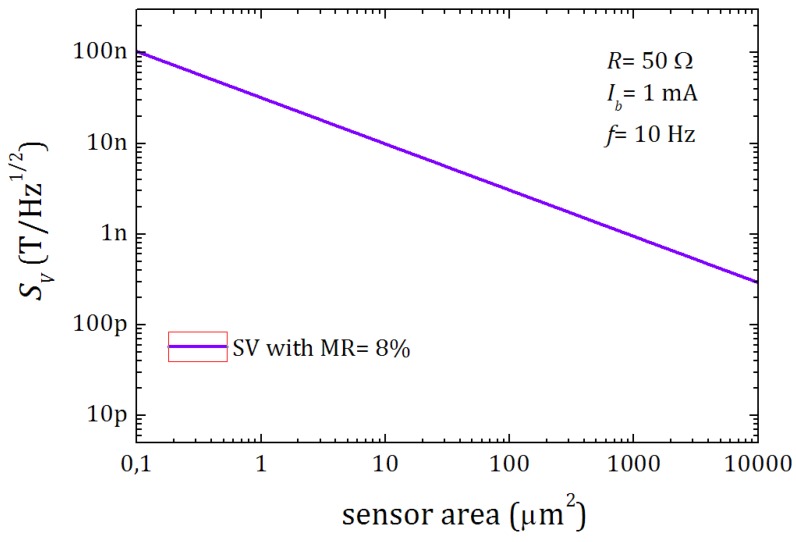
Analytical simulation of the detectivity $S_{V}\left(T/\sqrt{\mathrm{Hz}}\right)$ at 10Hz as a function of the magnetoresistance and sensor area for a spin valve [36].

The limit of detection of a sensor is conditioned by its signal to noise ratio (SNR). Because the noise is also amplified with the signal, a good sensitivity is not sufficient, but also a low electronic noise. Because magnetoresistance sensors such as spin-valves are mainly affected 1/f noise in the usual ranges of application, this noise is minimized when the operating frequency is high enough. Being the noise an statistical mechanism, it decreases as the size of the sensor increases. In Figure 8 we show the theoretical detectivity $S_{V}\left(T/\sqrt{\mathrm{Hz}}\right)$ at 10 Hz as a function of the magnetoresistance and sensor area for spin valve sensors, as reported in [36].

**Figure 9:**
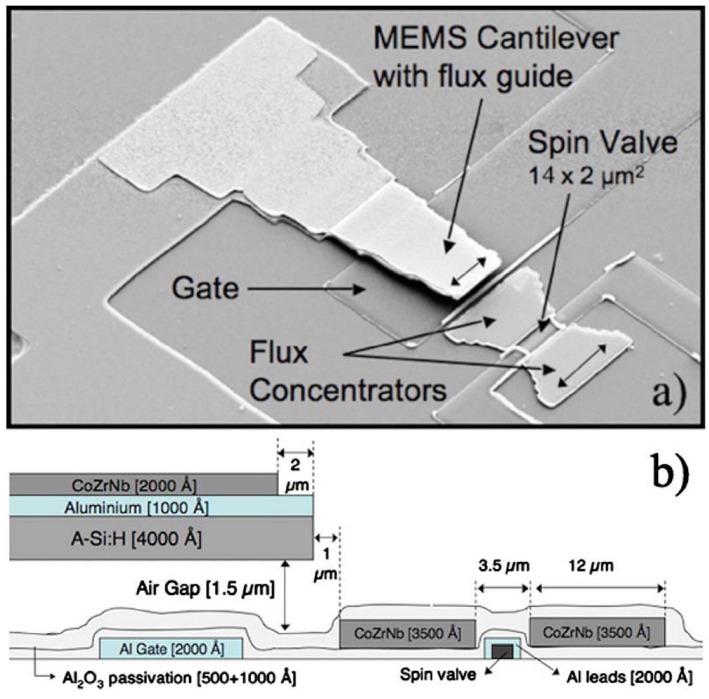
(**a**) SEM micrograph of the fabricated sensor with all its components: the MEMS cantilever with a 200 nm thick magnetic flux guide on top, the SV sensor (oscillating frequency: 400 kHz); (**b**) Cross sectional view of the device with all relevant features. (Reprinted with permission from [40].)

For taking advantage of the noise reduction with the frequency, the measured field can be AC modulated [39]. While traditionally this idea has been implemented by applying superimposed AC magnetic fields, the utilization of microelectromechanic systems (MEMS) together with the GMR technology has been approached. In this sense, MEMS resonators with incorporated MFC can be used to mechanically modulate static magnetic fields into the high frequency regime, where they can be sensed by a MR element, as reported in [40] and depicted in Figure 9. This is a major improvement to this technology allowing the detection of DC static fields down to 515 nT/$\sqrt{\mathrm{Hz}}$ as well as low frequency magnetic fields. More recent experiments show enhanced detectivity on the pT range [41].

## 4. Spreading the Range of Application

Integrating other elements with GMR sensors allows to confer additional capabilities to the final system as, for example, indirect measurement of magnetic related magnitudes, multiple sensing platforms or energy harvesting support.

### 4.1. Integration with Other Electrical and Electronic Elements

#### 4.1.1. Micro-Strips

**Figure 10:**
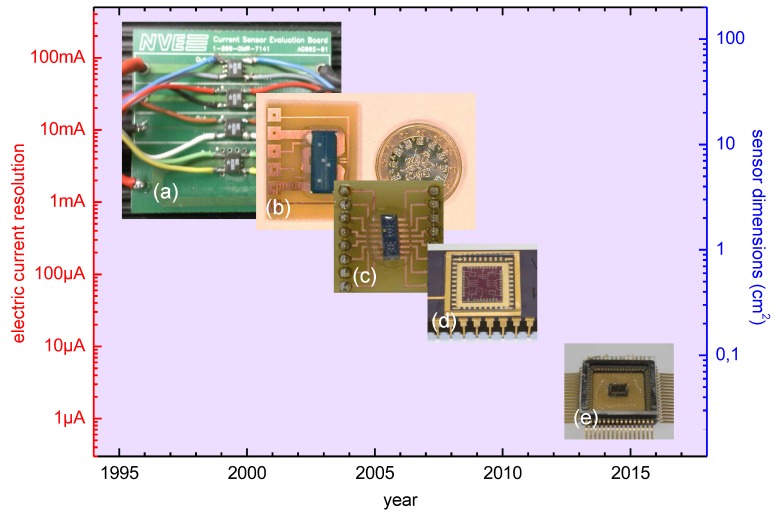
Historical evolution of GMR based current sensors regarding size and current resolution: (**a**) NVE evaluation board; (**b**) current sensor from [34]; (**c**) current sensor from [43]; (**d**) current sensor from [11]; (**e**) current sensor from [29].

GMR sensors are magnetic sensors and, hence, strips, often arranged as coils are commonly required as supporting elements. The integration of current (micro)strips with GMR sensors is directly required for the indirect measurement of electric current from its generated magnetic field [42]. Such approach was early proposed by NonVolatile Electronics for printed board circuits (PCB) current monitoring by means of general purpose GMR magnetic field sensors. These sensors were soldered to the PCB onto the conductive strap, and the electric current was indirectly measured from the generated magnetic field, as depicted in Figure 10a. First compact (non monolithic) dedicated GMR based current sensor is reported in [34]. Meandered current straps were engineered onto a small PCB piece. In parallel, a spin-valve based full Wheatstone bridge was designed onto a Si/SiO${\;}_{2}$ substrate, fitting the dimensions of the PCB current path. The whole system was wire bonded and sealed with silicone (see Figure 10b). In this way, electric currents up to 10 A were measured. Once the concept was demonstrated, the current strips were integrated within the fabrication process, requiring five lithographic steps, as detailed in [43] and shown in Figure 10c. Electric currents in the order of few mA were monitored in this way. This design was optimized in [11] (see Figure 10d). In this case, different geometries were tested and optimizing, resulting in measured currents lower than 1 mA. Such detection limit can be lowered to few *μ*A with the integration of the sensors with proper electronics [30] (see Figure 10e).

#### 4.1.2. Micro-Coils

The integration of coils and micro-coils with GMR sensors has been approached with two main objectives: as exciting field coils in eddy current testing (ECT) [44] applications and as magnetic tags attractors in bio-sensing [45].

**Figure 11:**
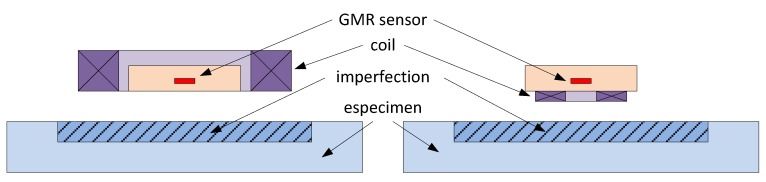
Possible geometric arrangements of coils with GMR sensors for ECT applications.

A basic ECT probe consists of a planar meander coil (exciting coil) and a magnetic sensor (GMR in our consideration), with different possible geometric arrangements, as depicted in Figure 11. The required moderate-to-high currents are a limitation, and integration at the PCB level is commonly preferred, in both configurations displayed in Figure 11a [46,47] and Figure 11b [48].

Magnetic beads are nowadays commonly used in microfluidic devices for labelling bio-analytes. Superparamagnetic-type magnetic beads only display magnetic moment when externally, so enabling an external field to control the beads remotely. In this sense, microcoils are used for attracting, trapping and guiding magnetic beads towards the GMR sensor. Such microcoils can be implemented as an integrated circuit (less than 1 mm${\;}^{2}$, less than 100 mA driving current) together with the GMR sensors in a square [49] or ring [50] shape.

#### 4.1.3. Antennas

Monolithically integrated radio frequency (RF) antennas together with GMR sensors are demonstrated in [51]. A three turn loop antenna is engineered on a metal layer with a constriction close to a spin-valve based magnetoresistor. The sensor is biased through the loop antenna as well as sensing the generated magnetic field. By harmonic analysis considerations, information from the field can be extracted. With this approach, fields below 3 μT (15 dBm at the RF source) at 130 MHz were detected. The capability of the antenna for self-powering the sensor was then analysed in [52].

#### 4.1.4. Hall Sensors

GMR sensors display notably better sensitivities than Hall sensors. Because the axis of sensitivity of GMR sensors is parallel to the layers’ planes and it is perpendicular to its surface in Hall sensors, they can be used together for obtaining multi-dimensional sensing. In [53], a combination of an array of GMR sensors with an array of Hall sensors is proposed to measure all three components of magnetic vectors and visualize the magnetic field distribution on a 2-D plane. An spatial resolution of 0.78 mm in the *z*-direction (with 1024 Hall sensors) and 2.34 mm in the *x*-direction (with 100 GMR sensors) is reported, imaging at 6 fps. An improved approach is presented in [54] for inspection of inclusions in cold-rolled strips by means of magnetic leakage testing using linearly integrated Hall and GMR sensors arrays. The spatial resolution of the sensors was 0.52 mm.

### 4.2. Microfluidics

The concept “Lab-on-chip” emerged due to the real chance of integrating microfluidic structures (including channels, pumps, reservoirs, mixers ...) with sensors and electronics. In this scenario, microfluidic biosensing systems using magnetic nanoparticles have demonstrated their potentiality in the detection of different analytes including molecules and cells [55]. Focusing on GMR sensors, the major challenge is to develop low temperature (not much higher than 100 ${\;}^{\circ }$C) and non-aggressive processes (physical etching or lift-off), preventing the damage of the underlying GMR elements. For these low temperature applications, the use of polymers such as polymethyl methacrylate (PMMA), polydimethylsiloxane (PDMS), polyethylene terephtalate (PET), epoxies such as SU-8 or even standard photoresists is necessary.

**Figure 12:**
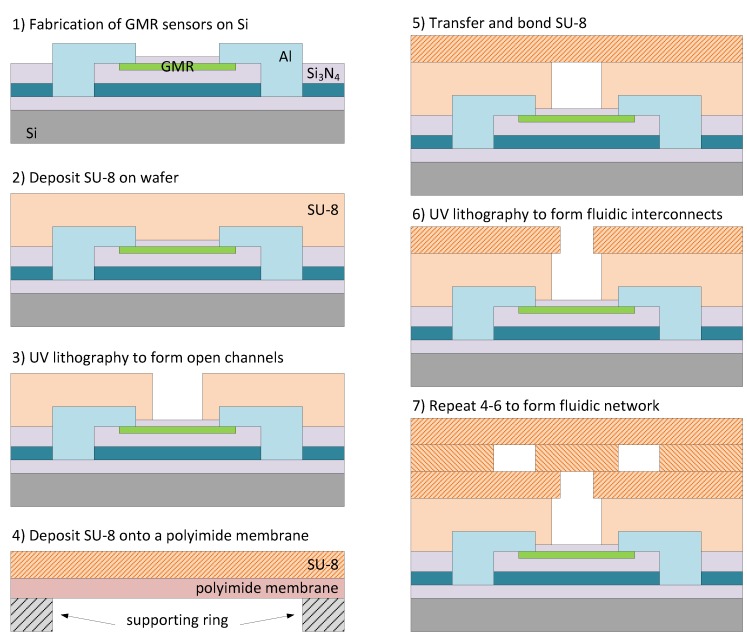
The process flow for integrating GMR sensors with 3-D SU-8 microfluidic system (as reported in [56]).

A pioneer work on integration of SU-8 microchannels onto a densely packed GMR platform (4” wafer) is reported in [56] and depicted in Figure 12. GMR devices were firstly sputtered, patterned and interconnected, and then passivated with Si${\;}_{3}$N${\;}_{4}$ (1). Then SU-8 was spin-coated directly on top of the microstructured wafer (2), with improved planarization. The SU-8 UV exposition and patterning process was aligned with the underlying sensors. Then, a non exposed SU-8 was spin-coated onto a polyimide membrane, peeled off, and transferred on top of the wafer (4 and 5). A thermal process was applied to enhance the bonding. This second SU-8 layer can be patterned (6). Steps (4) to (6) can be repeated as many times as the requirements of the microfluidics network. Microfluidics implemented onto non-photosensitive materials (PDMS) can be directly (mechanically) attached to the sensor wafer [57] or to a sensor array [58]. Fabrication of microfluidics channels have also successfully fabricated by making use of standard photoresist films [59].

From then, techniques and processes have been improved and the resulting devices have increased its complexity. In [60], a previously engineered molded interconnection device is used as the supporting element for the GMR/Si chip, also including the micro-channels. A flexible interconnect foil is attached to connect the chip with the read-out electronics. In [61], tapered current lines and current loops were also included for generating the exciting magnetic field for the micro-beads and guiding them to the sensing area.

**Figure 13:**
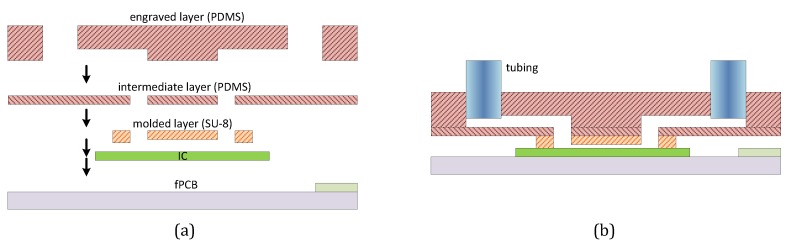
Hybrid soft lithography/laser engraved PDMS fabrication method (as reported in [62]).

An elaborated assembly process is reported in [62] in order to minimize the handicap do to the size mismatch between sensors ICs (in the range of mm${\;}^{2}$) and microfluidics systems (in the range of cm${\;}^{2}$) by integrating three separate pieces of PDMS, as depicted in Figure 13a. Firstly, a millimetre sized piece of PDMS with soft lithography defined channels was aligned and bonded directly to the IC surface. Then, a centimetre-size PDMS membrane with laser micromachined through-holes interlayer was designed. Finally, a centimetre-sized PDMS piece with laser engraved microchannels was fabricated. The separate pieces of PDMS were bonded together by stamping them into spin-coated uncured PDMS on a Si wafer, aligning the pieces together under a stereoscope with a mask aligner, and baking. The final result is shown in Figure 13b.

**Figure 14:**
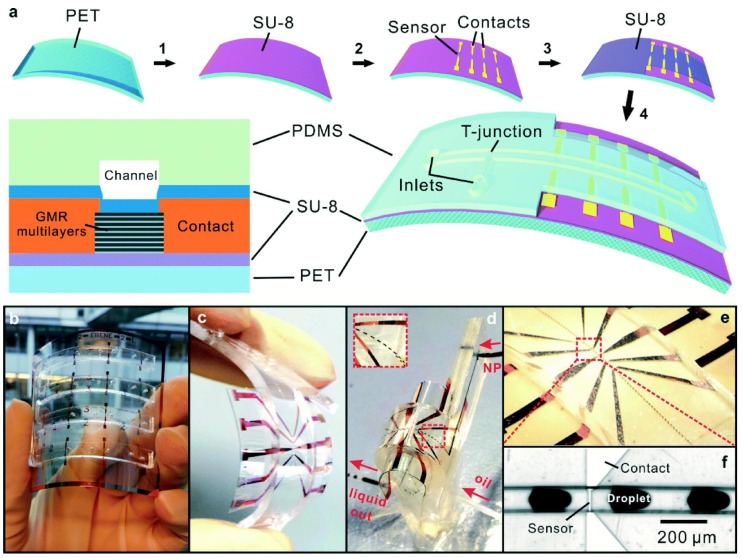
(**a**) Fabrication process of a flexible magnetoresistive analytic device; (**b**) electrodes integrated with microfluidic channels; (**c**) flexible analytical device; (**d**) device filled with liquid; (**e**) real time detection of emulsion droplets with magnetic nano-beads (Reprinted from [63]—Published by The Royal Society of Chemistry).

Recently, a full integration of GMR sensors with microfluidics into a flexible device has been reported in [63] and depicted in Figure 14. In this case, highly sensitive GMR [Py(1.5 nm)/Cu(2.3 nm)]${\;}_{30}$ multilayers (Py = Ni${\;}_{81}$Fe${\;}_{19}$) were deposited by magnetron sputtering onto a PET foil buffered with a SU-8 layer and patterned by lift-off. Contacts were also defined by lift-off. A SU-8 2 layer (700 nm) was used as passivation layer. The microfluidics channels were prepared in a PDMS layer deposited onto a Si substrate, and peeled off after the patterning. So the resulting microfluidics network was aligned with the chip and bonded with the help of a thermal process. The highest temperature achieved during the process was 200 ${\;}^{\circ }$C (during 5 min).

### 4.3. Full Systems

After achieving this level of maturity, full systems based on GMR sensors have been proposed in the recent past. An excellent example is found in [64], where the development of a magnetoresistive biochip portable platform is fully described. It includes a GMR sensor chip, placed onto a PCB, with a mechanically attached microfluidic channel, as a exchangeable card. Electronics are designed in parallel (including biasing, acquisition and communication circuits) and housed in a magnetically shielded box, with a hole for introducing the chip-card. This system was subsequently evolved (by utilizing ASICs) for developing a neuronal signal detector for biologically generated magnetic fields [65]. In a less sophisticated approach, a GMR-based ECT instrument for the detection and characterization of cracks in planar surfaces is proposed at a hand-held level as described in [66]. In this case, a standard computer mouse is used as centimetre sized housing box for the GMR sensor and the exciting coil.

Nowadays, the achieved level of integration has enhanced the compactness of the systems, so allowing the measurement of smaller magnetic fields in smaller volumes, and promoting the deployment of specific applications. Focusing on the the bio-world, based GMR systems have been successfully used for biomolecular recognition/detection, cell sorting/counting, single molecule actuation/detection or biomedical imaging [45]. We will comment on three successful cases reported on the last year (2015) in order to illustrate the maturity of the integration processes.

**Figure 15:**
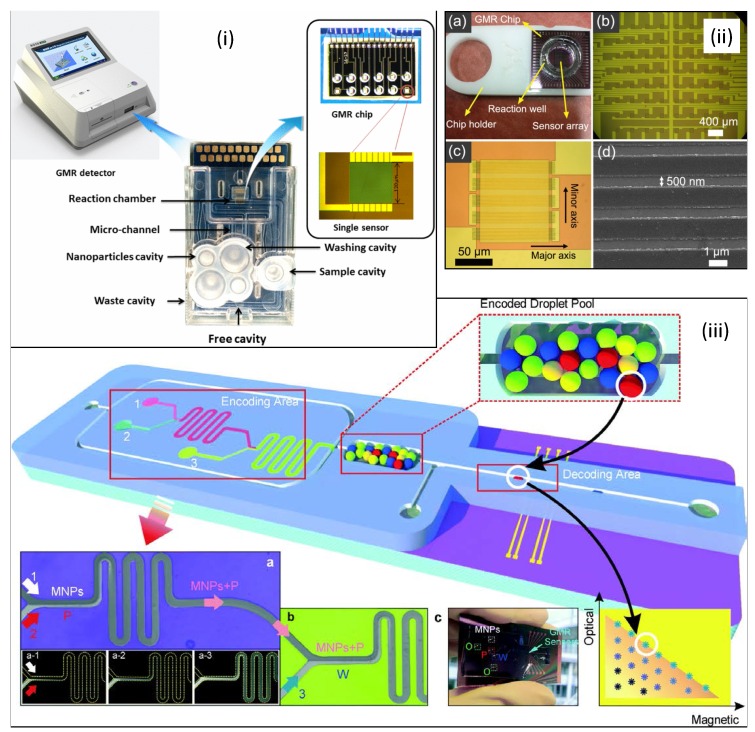
Full systems: (i) high-sensitivity cardiac multibiomarker detection system (reprinted with permission from [67]); (ii) biosensing probe station system for multiple protein assays [68]; (iii) Magnetofluidic platform for multidimensional magnetic and optical barcoding of droplets (Reprinted from [69]—Published by The Royal Society of Chemistry).

In [67] a novel high-sensitivity cardiac multibiomarker detection system based on a microfluidic chip and GMR sensors is presented. The considered multilayer structure was Al${\;}_{2}$O${\;}_{3}$ (40 nm) / NiFe (3 nm) / CoFe (1 nm) / Cu (2 nm) / CoFe (2 nm) / PtMn (10 nm) / Si (450 μm), arranged in 12 single sensors, each one composed of 75 linear elements (1 μm × 150 μm), with a resistance of 7 kΩ and a sensitivity of 14 Ω/G. The microfluidic system was fabricated with acrylonitrile butadiene styrene (ABS) plastic layer with a PDMS cover. It is depicted in Figure 15i. In this way, cardiac biomarkers NT-proBNP and cTnI could be detected simultaneously in 20 min, with limits of detection of 0.33 pg/ml and 1.43 pg/ml, with the help of 128 nm particles as magnetic tags.

A giant magnetoresistive-based biosensing probe station system for multiplex protein assays is described in [70]. The multilayer GMR film structure was designed as Ta/NiFe[20]/CoFe[10]/Cu[33]/CoFe[25]/IrMn[80]/Ta (units in Angstroms) and deposited by sputtering. An array of 8 × 8 sensors, each one consisting of 50 strips (120 μm × 0.75 μm). A reaction well of PMMA was attached onto the chip surface. It is depicted in Figure 15ii. The connection between the GMR sensor and the circuit board is accomplished by a probe station holding a pin array. With this systems, antigens such as PAPP-A, PCSK9 and ST2 were detected at levels from 0.04 to 400 ng/mL.

As a last example, a combined magneto-optical labelling of droplets strategy is approached by means of an integrated system including an encoding area, an encoded droplet pool and a magnetic decoding area, as detailed in Figure 15iii. Optical encoding is based on fluorescence and magnetic encoding is based on magnetic nanoparticles. GMR sensors are composed of a permalloy multilayer ([Py(1.5 nm)/Cu(2.3 nm)]${\;}_{30}$, Py = Ni${\;}_{81}$Fe${\;}_{19}$) structured into a meander composed of 19 turns of 3 × 100 μm${\;}^{2}$. PDMS and SU-8 are used as materials for the microfluidic network, which was attached by amine-epoxy chemistry.

## 5. Conclusions

GMR sensors have achieved their maturity in a unusually short period of time. In fact, the same groups that started on the deposition and characterization of primary GMR sensing structures are nowadays working in cutting-edge applications, within a period of 25 years. Responding to the requirements of last applications, mainly in bio-technology, the researchers have solved the integration of GMR sensors within a huge list of devices from different heterogeneous technologies: current strips, coils, magnetic flux guides, CMOS standard microelectronics, Hall sensors or microfluidics networks, through the definition of low temperature fabrication processes. The possibility of deposition onto flexible bio-compatible substrates has also been reported. Some of these full systems can incorporate more than one of these technologies, in a very compact fashion. In this way, GMR sensors have notably improved their response parameters, moving towards the pico-world. The detection of pT magnetic fields in sub-millimetre volumes has been demonstrated, widely spreading the range of the applications, mainly in bio-technology scenarios, where pL fluids can nowadays be managed.
